# Genotoxic Potential of Nanoparticles: Structural and Functional Modifications in DNA

**DOI:** 10.3389/fgene.2021.728250

**Published:** 2021-09-29

**Authors:** Ritesh K Shukla, Ashish Badiye, Kamayani Vajpayee, Neeti Kapoor

**Affiliations:** ^1^ Biological and Life Sciences, School of Arts and Sciences, Ahmedabad University, Ahmedabad, India; ^2^ Department of Forensic Science, Government Institute of Forensic Science, Nagpur, India

**Keywords:** nanoparticles, DNA, DNA damage, DNA repair, DNA methylation, genotoxic, structural and functional modifications

## Abstract

The rapid advancement of nanotechnology enhances the production of different nanoparticles that meet the demand of various fields like biomedical sciences, industrial, material sciences and biotechnology, etc. This technological development increases the chances of nanoparticles exposure to human beings, which can threaten their health. It is well known that various cellular processes (transcription, translation, and replication during cell proliferation, cell cycle, cell differentiation) in which genetic materials (DNA and RNA) are involved play a vital role to maintain any structural and functional modification into it. When nanoparticles come into the vicinity of the cellular system, chances of uptake become high due to their small size. This cellular uptake of nanoparticles enhances its interaction with DNA, leading to structural and functional modification (DNA damage/repair, DNA methylation) into the DNA. These modifications exhibit adverse effects on the cellular system, consequently showing its inadvertent effect on human health. Therefore, in the present study, an attempt has been made to elucidate the genotoxic mechanism of nanoparticles in the context of structural and functional modifications of DNA.

## Introduction

In recent years, Nanotechnology has reached an advanced level with significant research efforts. It is one of the fastest-growing fields with broad applications in almost every sector: health care and medicine, cosmetics, material science, food science, IT sector, etc. (Table 1). In 1959, **Richard P. Feynman** introduced nanotechnology in his famous talk, “***there is plenty of room at the bottom***.” Later, in 1965 he won the Nobel prize for his revolutionary invention of Nanotechnology. Since then, several inventions in natural science have harnessed the multi-functionality of the particles at nanoscale (Sanchez and Sobolev, 2010).

**TABLE 1:** The broad application of commonly used nanoparticles (NPs).

Sector	NPs	Application	References
Health and medicine	Au NPs, Al NPs, Cu NPs, and semiconductors such as Quantum dots, carbon nanotube	Cancer therapy	Rudramurthy and Swamy, (2018)
	Quantum dots	Drug delivery cell targeting/imaging, fluorescent probes and diagnostics	
	CoO NPs	Antigen delivery system for antitumor vaccine	
	Ti, V, Cr, Re, Mn, Au, and Cu NPs	Photo ctivated chemotherapy in cancer	
	Au NPs, quantum dots, silica NPs, inorganic phosphor NPs	Detecting viruses, hormones, specific a, thyroid-stimulating factors, DNA	
	Ag NPs, MgO NPs, TiO_2_ NPs, ZnO NPs, Au NPs, Cu NPs, Bi NPs, carbon nanotubes	Antimicrobial activity	
Cosmetics	ZnO NPs, TiO_2_ NPs	Sun defence	Raj et al. (2012)
	Fullerenes and fullersomes	Age management and night wear lotions	
	Al NPs	Powder, concealer	
	Si NPs	Wrinkle aiding lotions	
Electronics	BaTiO3, Pb(Zr,Ti)O3, (Ba,Sr)-TiO3	Transducers, actuators, and high-K dielectrics	Allsopp et al. (2007)
	Carbon nanotubes	Semiconductors, additives to electrodes of lead-acid batteries, photovoltaic cells	
	ZnO NPs	Optoelectronic devices	
	GaN NPs	LEDs	
	quantum dot	Lasers, optical telecommunication, fuel cells	
	Platinum and platinum alloys NPs	Fuel cells	
	TiO2, CdSe nanoparticles	Photovoltaic devices	
Food industry	Magnetic oxide nanoparticles	detection of food-borne pathogens	Shafiq et al. (2020)
	Zinc oxide NPs	nanocomposite material for active packaging of food materials, food preservation	
	Silver nanoparticles, carbon-based graphene nanoplates	Food packaging	
	Titanium dioxide	White color enhancer in food products	
	Silver, gold, platinum, copper, zinc, and superparamagnetic nanoparticles	Detection of various toxins present in foodstuffs	
Forensic science	Gold nanoparticles	Latent fingerprint development, biosensor, PCR amplification, drug detection	Raijiwala et al., 2019
			Kapoor et al., 2019
			Pandya and Shukla 2018
			Lodha et al. (2013)
	Silver nanoparticles	Latent fingerprint development, biosensor, explosive detection	Raijiwala et al., 2019
			Arshad et al., 2019
			Pandya and Shukla (2018)
	Cu-ZnCdS quantum dot	Fingerprint and explosive detection	Wu et al. (2015)
	Curcumin NPs	Explosive detection	Pandya and Shukla (2018)
	Calcium carbonate nanoparticles	Document preservation	Pandya et al., 2019
			Kapoor et al. (2021)

**FIGURE 1:**
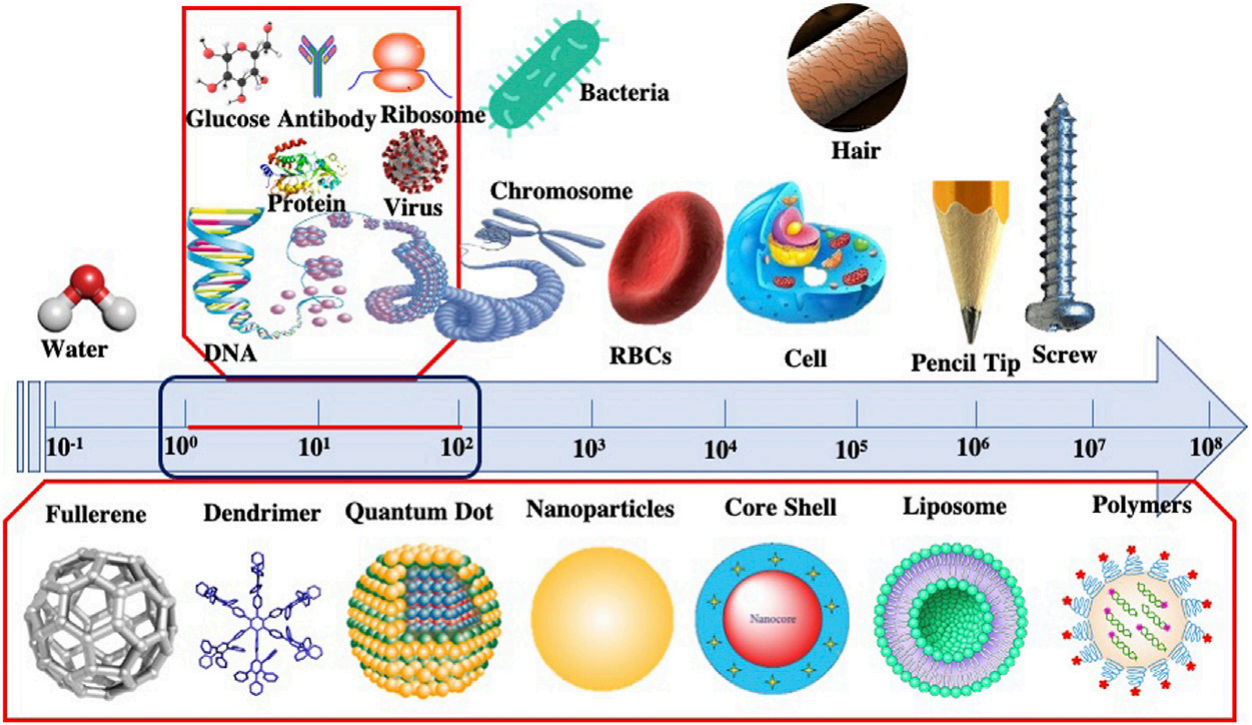
Comparative Size scale from macroscale to nanoscale represented by different objects.

Nanoparticles (NPs) can be defined as particles having size variations between 1 and 100 nm. Some researchers also call nanoparticles as ultrafine particles (Shown in Figure 1). At the nanoscale level, materials gain specific physicochemical properties which differ from their bulk or macro sizes. This nano-sized material yields a high surface to volume ratio, leading to an exponential increment in its reactivity, suitable for many scientific applications. Furthermore, the material’s mechanical, physical, biological, optical, and chemical characteristics vary significantly (López-Serrano et al., 2014). These distinctive properties drew much commercial interest and raised serious concern regarding the impact of nanoparticles on human health. Previous studies have demonstrated the adverse impacts of NPs on the environment and organisms, including Humans (AshaRani et al., 2008; Lovrić et al., 2005; (Oberdörster et al., 2007).

Humans and other species are continuously exposed to NPs, and the exposure is expected to increase in the coming years. The significant types of exposure include 1) external exposure, by the availability of NPs in the immediate surroundings of an organism and 2) internal exposure, referring to the particles taken up by the organism which can either interact with other biomolecules or be metabolized. The various routes depending upon the types discussed above can thus be *via* inhalation, occupational and cutaneous exposure (Griffitt et al., 2008; Maurer-Jones et al., 2013). The property of nanoparticles to interact with human biology holds a great promise in biomedical sciences (Table 2) but, on the other hand, poses a deleterious impact on human health, and genotoxicity is one of them.

**TABLE 2:** Application of NPs in medicine.

Nanoparticle (NPs)	Application	References
Silica nanoparticles	Nano structuring, drug delivery, and optical imaging agents	Jeelani et al. (2019)
Carbon nanoparticles	Early detection of cancer cells, and can act as markers in imaging diagnostics	Lisik and Krokosz, (2021)
Titanium dioxide	Psoriasis, cancer (treatment of malignant tumors)	Ziental et al. (2020)
Magnetic nanoparticles	Image-enhancing agents in MRI and magnetic particle imaging (MPI), immunoassays, cancer treatment, drug delivery and gene therapy agents, brain cancer treatment	Stueber et al. (2021)
Zinc oxide	Antibacterial, antifungal, antiviral, and anti-cancer	Wiesmann et al. (2020)
Gold nanoparticles	Drug delivery, cancer diagnostics, bio-Imaging, antibacterial, antifungal and antiviral agents	Aghebati-Maleki et al. (2019)
		Klębowski et al. (2018)
Silver nanoparticles	Cancer diagnostics	Chugh et al. (2018)

Genotoxicity generally signifies the toxic effect against the genetic material of an individual. This may lead to permanent inheritable changes in genetic materials (mutagenicity) or even induce unregulated cell growth (Carcinogenicity) (Phillips and Arlt, 2009). Therefore, genotoxicity is considered an essential facet of carcinogenesis. The widely accepted mechanism of genotoxicity by NPs is the oxidative damage posed by reactive oxygen species (ROS) and reactive nitrogen species (RNS) (Wan et al., 2012). Thus, a brief understanding of molecular mechanisms behind the biological effects, especially the genotoxicity of NPs, is necessary. The establishment of standardized assays for the utilization of nanomaterials are also the need of the hour.

## Mechanisms Behind Genotoxicity

### Clastogenic Mechanisms

Clastogens are the chemical mutagens that have the property to cause DNA strand breaks. These, if not repaired or misrepaired, may result in the formation of an acentric chromosome. Several chemicals act as clastogens like acridine yellow, benzene and arsenic. Thus, the clastogenic mechanism of a clastogen could be categorized into direct mechanisms and indirect mechanisms.

#### Direct Clastogenic Mechanisms

Direct clastogenic mechanisms uphold various DNA lesions, like base oxidization fairly producing 8-hydroxy, 20 deoxyguanosine (8-OHdG), base nitration through RNS, methylation, oxidative deamination/depurination, producing apurinic sites, ring-opening, and finally, single strand breakages (SSB) and double-strand breakages (DSB) by inducing ring-opening (Fenech et al., 2020). However, these mechanisms can also have carcinogenic consequences if the mutations ensuing from oxidative DNA damage (base-pair mutations, deletions, and insertions)affect and result in oncogene activation and inactivation of tumour suppressor gene.

#### Indirect Clastogenic Mechanism

Indirect clastogenic mechanism induces preliminary lipid peroxidation *via* ROSThis causes the mediation of electrophilic a,b-unsaturated aldehydes like malondialdehyde (MDA) and 4-hydroxy-2-nonenal (4-HNE) in the production of exocyclic DNA adducts such as ethanol and propane adducts.

### Aneugenic Mechanisms

The aneugens are the genotoxic agents which act essentially on non-DNA targets such as spindle fibres, resulting in disruptions in the cell division cycle. This ultimately leads to abnormal chromosomal segregation. The mechanism entails nitration by RNS/ROS induced oxidative protein lesions like cysteine oxidation or nitrotyrosination. These lesions interact with the mitotic apparatus’s constituents such as achromatic spindles, microtubule-organizing centres, kinetochores and spawn the disruption of chromosome segregation and migration during equatorial mitosis arrangement chromosomal loss due to non-disjunction in the anaphase (Parry, 2002).

### Production of DNA Adducts

DNA Adducts are the products formed when a carcinogen gets covalently attached to a DNA moiety, thus activating carcinogenesis. The number of DNA adducts thus formed in a cell can be used as a marker for genotoxicity. Neutrophils readily absorb Polycyclic Aromatic Hydrocarbons (PAH) and deliver them to the cytosol. These metabolites thus interact with DNA and associated proteins to form bulky DNA adducts. As a result, PAH bioactivation occurs, which results in the concentration of intercellular genotoxic electrophilic molecules. Further, bioactivation of these PAH molecules generates ROS/RNS, which ultimately affects the nucleotide excision repair mechanism (Güngör et al., 2007).

## Detection and Assessment of Genotoxicity

**FIGURE 2:**
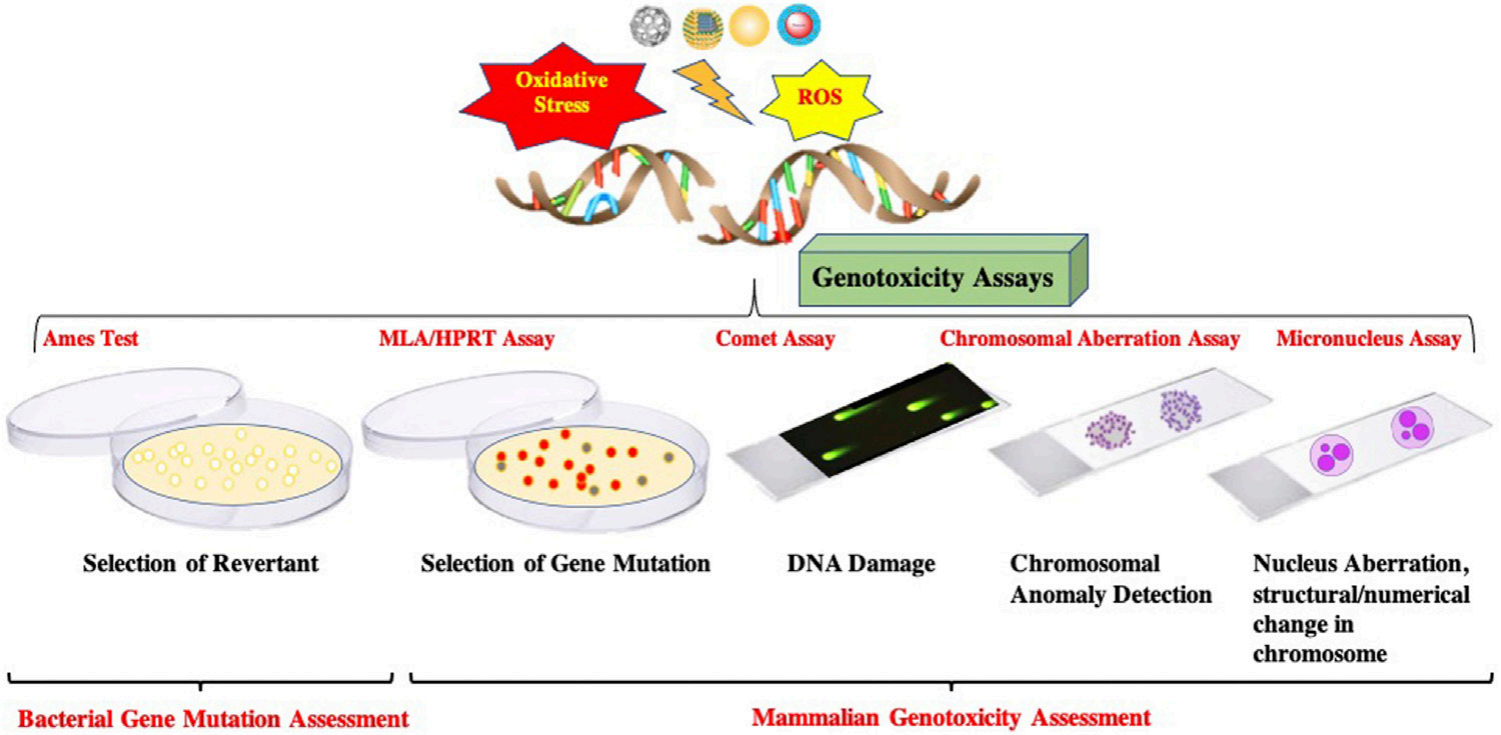
Schematic representation of standard battery of Genotoxicity Assays.

Genotoxicity can be assessed using a standard battery of assays and protocols that elucidate the gene mutation and DNA damage (Shown in Figure 2). In addition, these assays can identify/measure different types of genotoxic effects. Thus, the following section delineates battery standard assays performed presently to access the genotoxicity.

### Gene Mutations

Gene mutations can either be point mutations or frameshift mutations where several base pairs can be observed. The traditional assays detecting gene mutations are the Ames test, HPRT test, the mouse lymphoma test and the use of transgenic mouse strains.

#### Ames Test

The Ames test infers the gene mutations in various strains of *Salmonella typhimurium*. The bacteria used in this test carry a mutation in their genome containing operon for histidine synthesis. The presence of a genotoxic substance causes a reverse mutation in the auxotrophic bacteria converting them to prototrophic ones. The bacterial strain TA102 is frequently used to measure oxidative lesions. Several other such strains are employed for assessing different genotoxic effects. The Ames has its limitations in detecting clastogenic or aneugenic chromosome mutations. Nevertheless, it proves to be a good tool for detecting gene mutations caused either by direct or indirect genotoxic agents.

#### HPRT Assay

In the **hypoxanthine-guanine phosphoribosyltransferase (HPRT)** assay, gene mutations at the HPRT locus in the V79 cell lines of Chinese hamster pulmonary fibroblasts or Chinese hamster ovary (CHO) cell lines are detected. The test is primarily based upon the enzymatic property of HPRT, which mediates the phosphoribosylation of 6-thioguanine (6-TG). Thus, the mutation at the HPRT gene is evaluated by counting clones that are resistant to 6-thioguanine.

#### Mouse Lymphoma Assay

**The mouse lymphoma assay** utilizes the L5178Y mouse lymphoma cell line. This cell line is heterozygous for the thymidine kinase locus (tk+/-). The tk+ allele is deactivated in this test, resulting in loss of heterozygosity and resistance to toxic base analogue trifluorothymidine. Moreover, it allows the selection of tk-cell populations. The test can thus be utilized to detect clastogenic/aneugenic chromosome mutations along with gene mutations.

**Transgenic mice strains** are readily being used as a traditional *in-vivo* method to evaluate genotoxicity. Several transgenic mice models in which one gene of interest are inactivated and subsequent studies related to the genotoxic substance. Examples include the BigBlue model and MutaMouse.

### Detection of Primary DNA Alterations

The standard assays used to detect Primary DNA alterations include the Comet assay and the unscheduled DNA synthesis test (UDS).

#### Comet Assay

The Comet assay is also known as **single-cell gel electrophoresis**. It is a simple, rapid and sensitive technique to detect DNA single/double-strand breaks, alkali-labile sites, and cross-linking sites in a single individual cell. Initially, it was developed to work under neutral conditions, but researchers modified it to work well under an alkaline environment to evaluate low levels of DNA strand breaks as the science progressed. The Comet assay works on the electrokinetic movement of the negatively charged DNA fragments in an agarose gel. Here the extent of DNA migration corresponds to the amount of DNA damage. In Comet assay, the cell suspension is added to low melting point agarose. The above mixture is then spread evenly onto the microscopic glass slide precoated with normal melting agarose. The cell lysis occurs with the help of detergents, and a specific pH is maintained for DNA unwinding. At a neutral pH, double-strand breaks and cross-links become feasible, whereas single and double-strand breaks, incomplete excision repair sites and cross-links can be detected at slightly high pH (12.1–12.4). Alkali labile sites can be detected at a pH ranging higher than 12.6.

After DNA unwinding, an electric field is applied onto the microscopic slide containing DNA; the fragmented DNA moves out of the cell and migrates towards the positively charged electrode. The slides are stained with DNA binding dyes (Ethidium bromide, propidium iodide) where the migration of fragmented DNA appears like a Comet structure in which the head portion comprises intact tail is formed of damaged/fragmented DNA. The size, shape and DNA distribution positively correlates to the extent of DNA damage. Comet assay is combined with several bacterial enzymes like endonuclease III, formamidopyrimidine glycosylase (fpg), etc. Thus, the comet assay is a robust technique to identify oxidative DNA damage at the cellular level. The technique also becomes advantageous since a minimal sample quantity is required for the detection (Shukla 2017).

#### Unscheduled DNA Synthesis

The UDS test identifies the lesions while measuring the intensity of DNA synthesis (S-phase) during the repair process. In the UDS technique, the cell lines are exposed to a particular chemical followed by cultivating them in the presence of tritium labelled thymidine (^3^H-TdR). If any DNA damage has occurred, the cell recruits DNA repair proteins that incorporate tritium labelled thymidine during the repairing process. Thus, this radioactive thymidine can be measured directly to the amount of DNA damage using autoradiography.

#### SMART Assay in *D. melanogaster*

The **SMART** (Somatic Mutation and Recombination Tests) Assay of *Drosophila melanogaster* measures the toxicological effects of a given chemical as a function of mutation leading to the alteration of standard life functions and traits like sex ratio, number of eclosed individuals, developmental time, body size, growth, fertility, etc. In this assay, *D. melanogaster* is exposed to the substance/chemical studied, resulting in altered functions. Since *D. melanogaster* shares several metabolic pathways (DNA repair systems, digestion and absorption, etc.) analogous to mammals, the organism is preferred for toxicological studies. Designed in the 1980s, the two significant assays-wing spot test and eyespot test are currently being used to assess the genotoxic impact of the substance under study. In these assays, the *Drosophila* species are exposed to the substance in question leading to DNA damage/genetic alterations. These genetic alterations can then be phenotypically observed in the next generation (adults) and accessed through the wing spot and/or eyespot test. In an individual heterozygous for a trait, the loss of heterozygosity is phenotypically measured, allowing the quantification of DNA damage occurred (Soloneski and Larramendy, 2021).

### Chromosomal Mutations

Chromosomal mutations can be either clastogenic or aneugenic. Micronucleus assay (MN assay) is traditionally used to investigate chromosomal mutations or aberrations. Micronuclei are the fragments of the nucleus produced during a cell division. They are constituted from either acentric chromosomes or chromosome fragments caused by some clastogenic or aneugenic events. The MN assay is often performed along with Fluorescence *in situ* hybridization (FISH)technique or combined with CENP-A (centromere protein A) to specify whether the genotoxic effect is clastogenic or aneugenic. The micronucleus test can be utilized in *in-vivo*, *ex-vivo* and *in-vitro* studies.

## Genotoxic Mechanisms of NanoParticles

Studies on particle genotoxicity have long been a part of the research, ever since Doll first reported the carcinogenic effects of asbestos in 1955. Subsequently, research into particles genotoxicity and its possible mechanisms have seen a paradigm shift. Scholars have classified the mechanism of genotoxicity into two significant groups, **Primary genotoxicity and Secondary genotoxicity**. Further, the primary mechanisms are divided into **Direct effects** and **Indirect effects**.

Primary genotoxicity refers to the evocation of genetic damage without any inflammatory reaction. The direct primary effects are evoked by the mechanisms that involve the particle’s direct interplay with the genetic material and associated proteins. When particles contact the cellular system, they readily diffuse through nuclear membranes or physical/chemical interference during the cell cycle and directly interact with genomic DNA, producing ROS/RNS species. (Fubini 1998; Gonzalez et al., 2008). These ROS/RNS species produce free radicals that can directly affect DNA and indirectly affect the enhanced production of free radicals by mitochondria and membrane-bound NADPH oxidases and cause oxidative stress that leads to DNA damage. At the same time, secondary genotoxicity effects are thought to be associated with inflammatory cells (macrophages and PMN). The inflammatory reactions recruit these cells to initiate innate immunity, but this mechanism also evolves reactive free radicals, ultimately damaging the DNA (Azad et al., 2008).

In the case of nanoparticles, to project primary genotoxicity, the particle requires to enter the cytosol or the nuclear membrane and interact directly with DNA and associated proteins. Several studies have successfully reported the size-dependent entry of NPs and accumulation outside of endosomes and within specific cellular compartments (Tkachenko et al., 2003; Nativo et al., 2008; Chen and Vonmikecz, 2005). In addition, two studies have also demonstrated that the NPs can diffuse into the nuclear membrane and directly interact with the DNA (Li et al., 2003; Geiser et al., 2005).

Evidence from the studies also suggests a positive indirect primary genotoxic effect of the nanoparticles (Park et al., 2008; Lee et al., 2009; Wang et al., 2009). Various metal NPs like ZnO NPs, SiO_2_ NPs, TiO_2_ NPs have been shown to evoke the production of ROS and RNS to cause oxidative DNA damage (Shukla et al., 2011).

Robinson (2008) has studied and reported the secondary mechanism exhibited by nanoparticles. He suggested that the activation of the innate immunity reaction by phagocytes increases cellular oxygen consumption and thus results in the release of O^.-^, H_2_O_2_due to activation of the NADPH-oxidase system.

**FIGURE 3:**
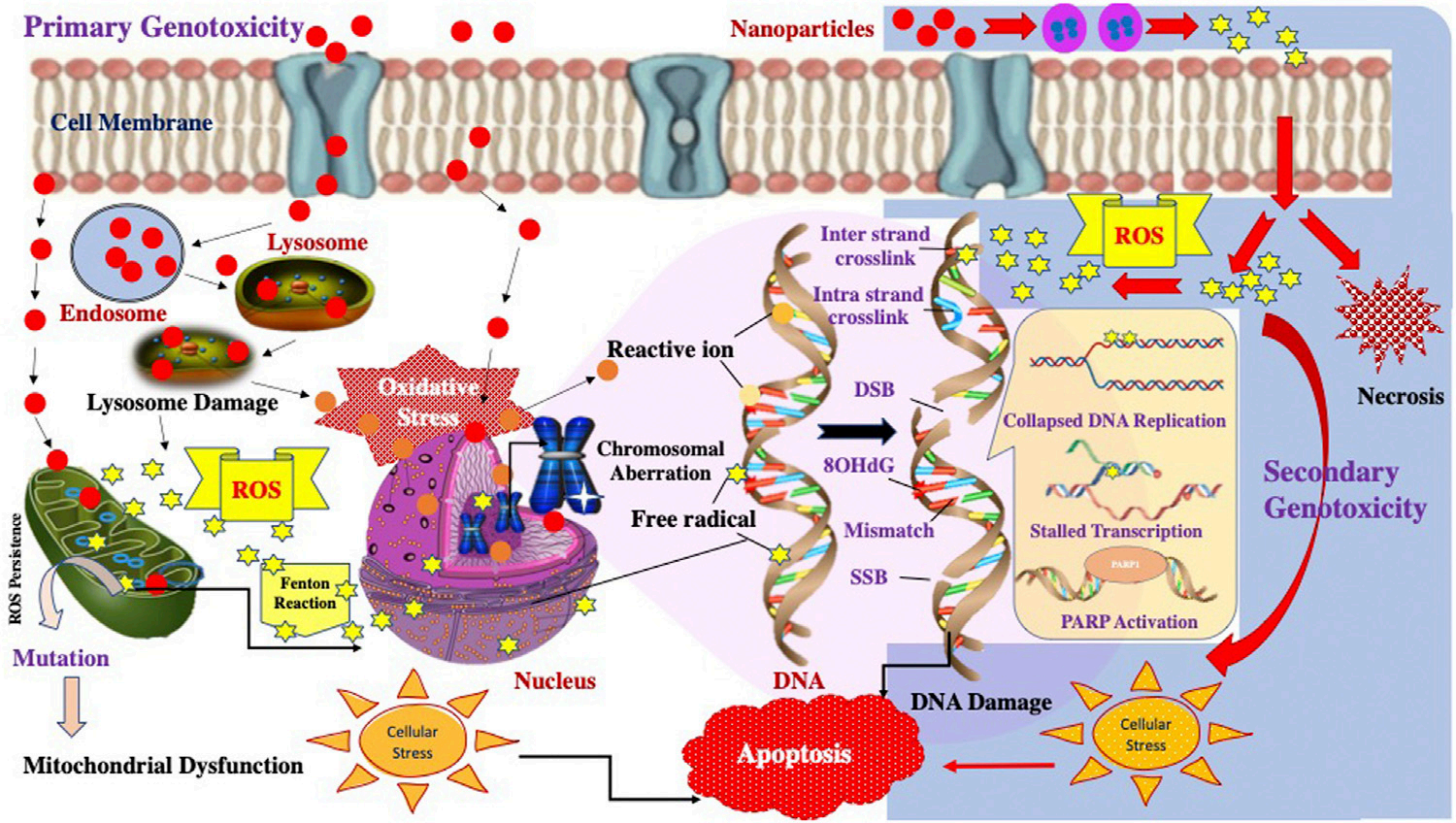
Schematic representation of the possible Primary and Secondary Genotoxicity mechanisms induced by nanoparticles.

Hence, Researchers have majorly drawn out three primary hypotheses behind the genotoxic mechanisms, which include 1) involvement of the surface effect; 2) Release of ROS/RNS due to the action of released transition metal ions by NPs, 3) activation of membrane receptors like epidermal growth factor receptor by transition metals. Figure 3 demonstrates the possible mechanism of genotoxicity posed by nanoparticles.

## Genotoxic Effects of Nanoparticles

The previous sections have drawn out a basic scheme of the mechanism underlying the genotoxicity of nanoparticles. Since it is now known that the nanoparticles induce DNA damage in several ways, it becomes crucial to understand how these NPs interact with the DNA and its associated set of proteins to hinder the repair mechanism and cause DNA damage finally. It also becomes essential to evaluate its impact on the epigenome of an organism to acknowledge how the gene expressions are being affected by these NPs.

### Impact on DNA Damage and Repair Processes

Several studies have explicitly reported that DNA damage is caused either by the direct interaction of NPs with the DNA or secondarily by initiating ROS/RNS mechanism in the cell, which leads to producing oxidized DNA bases (8-oxo-dGuo), single-strand breaks (SSBs), double-strand breaks (DSBs) and apurinic (AP) sites. Since DNA is prone to several mutagens, organisms have evolved with various routes of DNA repair systems to maintain the balance. DNA damage occurs when this “balance” situation is hindered by either excessive free radicals or malfunctioning repair systems.

The various repair systems are; 1) nucleotide excision repair, 2) base excision repair, 3) non-homologous end joining, 4) homologous recombination and 5) mismatch repair. While working with genotoxicity assays (micronuclei assay, Ames test, etc.), scholars have reported the effect of NPs on DNA repair systems. These studies have shown that the increased frequency of DNA lesions and breaks is DNA damage mainly caused by impaired (imbalanced) DNA repair systems. To analyze the DNA repair from an impairment, researchers have measured the activity of DNA repair systems, the intracellular content of DNA repair proteins and the expression of the genes that encode DNA repair proteins. For example, Demir et al. (2014) used DNA repair kinetic to probe ZnO-NP interaction with DNA repair systems. They observed an altered repair system in the exposed cells. In a similar study, with cells exposed to silver (Ag)-NPs, direct impairment of DNA repair processes was observed (Kruszewski et al., 2013).

A classification of the NPs into various groups has established in the EU-funded FP7 project named MARINA in 2015 (Arts et al., 2015), in which the interaction of each group of NPs with repair proteins was studied as follows:

#### Impact of Transition Metals Based NPs on the Function of DNA Repair Protein

Metal-based NPs like Ag-NPs, CuO-NPs, ZnO-NPs tend to release their metallic ions while they get dissolved in the cytoplasm. Previous studies revealed that the interaction of these metal ions is the primary cause of genotoxicity. These metals ions often have low mutagenicity. Moreover, their carcinogenicity is believed to affect the DNA repair process. This impact on DNA repair processes is directly linked to the type of metal from which the NPs are composed (Hartwig and Schwerdtle, 2002). For example, metals such as arsenic, cobalt and cadmium impair the DNA repair process by affecting BER and NER. When present in the cytosol NPs are subjected to acidic pH, where they release metal ions. In the vicinity of the cytosol, some proteins require metal as co-factors for their activation. These metals interact with such proteins and cause protein remodelling.

NPs can also alter the “metal haemostasis” of the cell leading to alterations in all the essential cellular processes. Zinc Fingers (ZF) pose a great example in this regard. ZF is required to interact with the DNA and RNA in the cell. The proteins have Zn (II) ions attached with the chain donors of histidines or cysteines. Upon exposure to Zn NPs, metal ions are released and replace these Zn (II), thereby distorting the 3-D structure of the protein’s active site, resulting in the inactivation of protein (Lebrun et al., 2014).

#### Impact of Active Nanoparticles on DNA Repair Proteins

The active class of NPs, as defined by Arts et al. (2015), are known to activate or inactivate biological molecules and other reactions due to their surface properties. For instance, Ce atoms at the surface of the CeO_2_NPs exists in +III and +IV oxidation states due to redox reactions on their surface. This redox reaction, in turn, leads to modifications in the surrounding molecules (Caputo et al., 2015). Similar is the case with TiO_2_ NPs, which tend to alter the DNA repair proteins due to their redox properties (Fenoglio et al., 2009).

#### Sequestration of DNA Repair Proteins in NP-Protein Corona

It is well known that the surface area to charge ratio of the NPs is very high. Due to this particular surface property, NPs tend to capture many molecules onto their surface, leading to the formation of what researchers call a “Nanoparticle Corona”. In addition, NPs are known for their high protein affinity. Therefore, when the NPs reach nucleoplasm, these proteins get caught into the corona leading to their inactivation. Further, due to the larger surface area of the NPs, they tend to “absorb” a large number of repair proteins leading to their deprivation. This sequestration of the limited proteins leads to the inactivation of the DNA repair system (Monopoli et al., 2011).

### Impact on Epigenome

**FIGURE 4:**
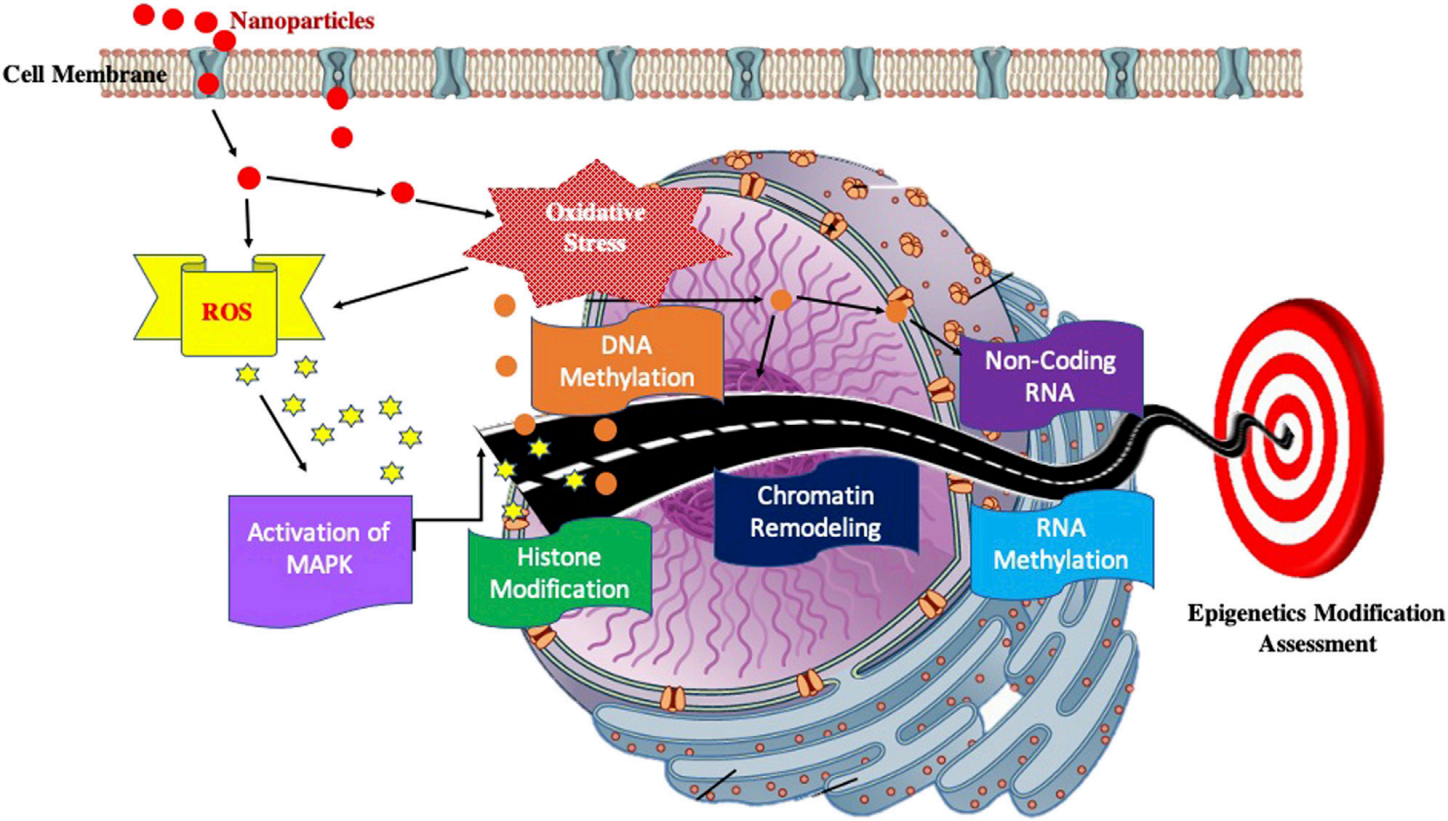
Block diagram showing possible pathway to elucidate the Impact on Epigenome.

It has not been so long when scientists have realized that deregulation of the epigenome of a cell leads to deleterious effects on an individual. Epigenetic modifications are stable and can be transmitted from one generation to another. This leads to a possibility that these deleterious changes/modifications may thus affect the next generation of the individual. The exposure of NPs and their impact on epigenome thus is a matter of concern. The epigenetic modifications can be elucidated by DNA modifications (methylation and hydroxymethylation), histone modification, chromatin remodelling, RNA methylation, and small and long non-coding RNAs (ncRNAs). (Figure 4).

#### Effect of Nanoparticles on DNA Methylation

DNA methylation is a phenomenon in which methyl groups are covalently bound to the C-5 position of the only cytosine base, followed by a guanine nucleotide (CpG island). This methyl binding affects the DNA accessibility by transcription factors and thus controls gene expression. If the promoter site of the gene is methylated, the gene is called switched-off and vice versa. The DNA methylation process is catalyzed by DNA methylases (DNMTs), and S- Adenyl Methionine (SAM) acts as a source for the methyl group. The DNMTs vary depending on their functions, such as DNMT3a, DNMT3b and DNMT1.

Several independent research groups have studied the impact of NPs, such as carbon-based SWCNTs/MWCNTs, TiO2, CuO, SiO2, etc., on DNA methylation (Lu et al., 2015a; Brown et al., 2015). These studies have identified that exposure to NPs cause locus-specific and global DNA hypomethylation. However, the extent of DNA modifications cannot be implicated from these studies. For example, Gong et al. (2010) exposed the human keratinocyte (HaCaT) cell lines to SiO_2_ NPs and observed a decrease of more than 20% in global DNA methylation. Here, SiO2 NPs had an essential role in reducing the levels of DNMTs. In a similar *in vivo* study, researchers reported Global DNA hypomethylation when examining lungs and blood of mice exposed to MWCNTs (Brown et al., 2015). In contrast to the previous study, HPLC-MS analysis of the “*in vitro*” study showed DNA hypermethylation (increase in DNA methylation) in human lung carcinoma cell line after exposure to MWCNTs (Li et al., 2016). In addition to this, researchers have also demonstrated that on exposure to permitted NPs on mammalian cells, both hyper and hypomethylation of LINE1 and Alu repeats were observed (Lu et al., 2015b). They also reported the downregulation of the DMNTs associated with NPs exposure.

Apart from studying the DNA methylation on repeated DNA sites, researchers have also studied the epigenetic changes caused by NPs at specific loci. Ha et al. (2015), while working with the *ALPL* gene in murine bone marrow stromal cells exposed to nano-hydroxyapatite, found a 40% increase in DNA methylation at the promoter region. It is evident from these studies that NPs tend to alter the DNA methylation marks over the genome, but their functional consequences are still unknown. Therefore, more study is required to elucidate the mechanism of NPs interaction with DNA and its effect on DNA methylation.

#### Effect of Nanoparticles on Histone Modifications

The post-translational modifications like acetylation, methylation, phosphorylation at the histone tails primarily affect the binding of the protein octamer to the DNA. These modifications either loosen up the DNA packaging or tighten them, ultimately controlling the gene expression. Not much has been explored about the effect of NPs on Histone modifications. Studies have shown that diffusion of NPs in the nucleus tempers various functions based upon the chromatin region affected. When interacting with the NPs, Heterochromatin causes shrinkage of the nucleus, whereas marginal modifications can be observed in euchromatin (Jennifer and Maciej, 2013).

Table 2A few studies have shown that histone modifications are also important targets for these NPs. For example, in one study with breast cancer cells exposed to cadmium telluride quantum dot treatment, chromatin condensation accompanied by global H3 hypoacetylation was observed. Interestingly, quantum dot exposures also lead to the expression of some apoptotic genes caused due to activation of p53 (Choi et al., 2007). Ag-NPs have also been studied for their effects on the epigenome. Researchers have found that they affect the enzymes involved in chromatin remodelling (Dubey et al., 2015).

#### Effect of Nanoparticles on Non-coding RNAs

Non-coding RNAs (ncRNAs) are broadly classified into short ncRNAs and long ncRNAs based upon their transcripts. These ncRNAs are associated with other epigenetic modifications like DNA methylation, histone post-translational modifications, chromatin remodelling. Therefore, they are sometimes the targets of the NPs, which tend to affect the process of DNA methylation and chromatin remodelling. A very handful of studies present the impact of the interaction between the ncRNAs and NPs.

Halappanavar et al. (2011) first reported the effect of surface-coated nano-titanium dioxide particles using a mouse model. They observed significant changes in the expression of 16 miRNAs in the mice lungs. In another such study, mice were exposed to carbon black nanoparticles where lungs and other organs of the mice were found to have altered miRNA expression. Further studies by Balansky et al. (2013), Nagano et al. (2013) and Eom et al. (2014) demonstrated the altered miRNA expressions.

Thus, *in vivo* and *in vitro* studies by several researchers (Table 3) provides evidence to the notion that these NPs tend to alter the epigenome in the following ways-

• Alteration of global and gene-specific DNA methylation (Pogribna and Hammons, 2021)
• Increase phosphorylation of histone H2AX accompanied by oxidative stress (Kopp et al., 2018)
• Increase the acetylation of histones H3 and H4 (Seidel et al., 2017)
• Increase the levels of HDAC2 protein whereas decrease the levels of HDAC1 and HDAC6 proteins (Seidel et al., 2017)

**TABLE 3:** *In vitro* and *in vivo* studies on impact of nanoparticles on DNA methylation.

Nanoparticles	Experiment model	Epigenetic effects	References
Carbon nanotubes (CNTs); single-walled and multi-walled carbon nanotubes (SWCNTs, MWCNTs)	Human THP-1 monocytes	No observed results in global DNA methylation (5-mC) or DNA hydroxymethylation (5-hmC)	Öner et al. (2016)
		Hypomethylation of 1,127 genes, including *STAT5A, JAK3*-*STAT6, VEGFA, NOTCH1, NOTCH4, NOSS, WNT5B, PRKCZ, SH2D2A, SFRP1, FGFR1, TF, NAP2K2, AKT1, MEIS1*	
SiO_2_-NPs	Human HaCaT cells	Global DNA hypomethylation	Gong et al. (2010)
		Dose-dependent decrease of the levels of DNMT1, DNMT3A, and methyl GpG binding protein 2 (MBD2)	
TiO_2_-NPs	Human small airway epithelial cells	Demethylation of SINE B1 repetitive elements	Lu et al. (2015a)
Au-NPs	Intratracheal administration to BALB/c mice	No effect on global DNA methylation and DNA hydroxymethylation	Tabish et al. (2017)
		Promotor hypermethylation in *Atm*, *Cdk*, and *Gsr* genes in mouse lung tissue	
		Promotor hypomethylation in *Gpx* gene in mouse lung tissue	
Ag-NPs	Human lung adenocarcinoma epithelial cells A549	Global DNA hypermethylation	Blanco et al. (2016)
CuO-NPs	Male BALB/c mice	Global DNA hypermethylation	Lu et al. (2015b)
		Reduced expression of DNA methyltrasferases, *Dnmt1*, *Dnmt3a*, and *DNMT3b*, and *Tet1*	

#### Inheritance and Restoration

Normally, an individual’s epigenome is well maintained and controlled (Pogribna and Hammons, 2021). However, upon the exposure of NPs and Nanomaterials, this epigenomic “balance” gets altered the expression profiles of coding and non-coding genes. The mechanism underlying this “un-balancing” remains unclear since there are several pathways involved in “controlling” the “balance”. Thus, alteration in a single pathway might not significantly cause the “un-balancing” of the epigenomic constitution, but a combined alteration in pathways (more than one) will eventually cause epigenomic aberration.

There have been studies to link the changes in DNA and histone modifications leading to aberration in the functioning of chromatin-modifying proteins. However, the results produced were inconclusive (Pogribna and Hammons, 2021). Further, a study by Valinluck (2004) states that the phenomenon of oxidative stress and formation of DNA lesion hinders the process of DNA methylation by interfering with the Dnmts, a cause of DNA hypomethylation. Another study suggests that exposure to NPs and nanomaterials leads to depleting glutathione-a significant contributor to DNA methylation and histone alterations (Deobagkar et al., 2016; Long et al., 2019). Few researchers have studied the effect of nanoparticles on histone modification which is shown in Table 4.

**TABLE 4:** *In vitro* and *in vivo* studies on the impact of nanoparticles on histone modification.

Nanoparticles	Experiment model	Epigenetic effects	References
ZnO-NPs	Human bladder cancer T24 cells	Decrease of global histone 3 lysine 27 trimethylation (H3K27me3) at the *RUNX3* gene promoter	Zhang et al. (2020)
CuO-NPs	Human A549 cells	Decrease of total HDAC activity	Kalaiarasi et al. (2017)
		Reduction in the levels of HDAC1, HDAC2, HDAC3, HDAC5, HDAC9, and HDAC11 mRNA transcripts	
Ag-NPs	Human A549, MCF7, and HaCat cells	Increase in histone 3 serine 10 phosphorylation (H3S10ph	Zhao et al. (2017)
As_2_O_3_-NPs	Human embryonic kidney (HEK) 293T	Decrease in global H4K16ac	Liu et al. (2015)
Au-NPs	Small airway epithelial cells	Decrease in H3K27me3	Shyamasundar et al. (2015)
TiO_2_-NPs	Human adipose delivered stem cells (hASCs)	Increase of H3K4 methylation at the promoter region of osteogenic genes RUNX2 and osteocalcin (OC)	Lv et al. (2015)
		Inhibition of histone demethylate	
		RBP2 expression	
SiO_2_-NPs	Human A549 cells	Decreased levels of SIRT6 histone deacetylase (HDAC) transcript and protein	Zhang et al. (2018)

The alterations or “un-balance” in epigenome leads to several diseases like cancer and others (Wu et al., 2020; Yang et al., 2020) in the respective individual. However, how these epigenetic alterations are restored in a cell remains unanswered. Further studies on the maintenance and restoration of the epigenome alteration need to be explored to get an insight into the impact of NPs over the subsequent generations.

The non-genetic inheritance pattern of the epigenome relies on parental contributions. If an altered gene is inherited, it will lead to the expression or repression of the gene affected in the progeny depending upon its imprinting status (maternally/paternally). However, researches on how these alterations are passed on to the generations are yet to be established.

## Case Studies

The present section delineates several *in vivo* and *in-vitro* studies that probe into the genotoxic effects of the NPs. For a better understanding, the nanoparticles have been classified broadly under carbon-based nanoparticles and metal oxide-based nanoparticles.

### Carbon-Based Nanoparticles

Carbon-based nanoparticles are found in various shapes and conformations like single-wall carbon nanotubes (SWCNTs), multi-walled carbon nanotubes (MWCNTs), and carbon fullerenes (C60 fullerenes).

#### Single Wall Carbon Nanotubes (SWCNTs)

Ema et al. (2012a)studied the genotoxic effects of SWCNTs (mean diameter- 1.8 nm and Brunauer-Emmett-Teller [BET] surface area of 878 m^2^ g^−1^ containing 4.4% iron). Various assays are used as a bacterial reverse mutation test (involving T histidine-requiring *Salmonella typhimurium* strains TA98, TA100, TA1535, and TA1357 and the tryptophan-requiring *Escherichia coli* mutant WP2uvrA), an *in vitro* mammalian chromosomal aberration test (The Chinese hamster lung fibroblast cell line CHL/IU) and a micronucleus test on mammalian erythrocytes. They did not observe any genotoxic effects on any of the assays performed. In another study by Kisin et al., 2007, the positive genotoxic effects (DNA double-strand break) by SWCNTs [diameters from 0.4 to 1.2 nm, length of 1–3 mm and surface area of 1,040 m^2^/g) and containing 99.7 wt% element carbon and iron levels of about 2.3 mg Fe/g sample (0.23 wt%)] through comet assay (Chinese hamster lung fibroblasts -V79 cell line) was confirmed. However, they did not observe any positive results in the micronucleus test and Ames assay (*Salmonella* strain YG1024 or YG1029).

Shvedova et al., 2008 in their study with C57BL/6 mice, investigated the genotoxicity (SWCNTs with diameters 0.8–1.2 nm, and length 100–1,000 nm). They observed positive genotoxicity by measuring mutations in the *k-ras* gene. Previous studies by Pacurari et al., 2008; Yang et al., 2009; Migliore et al., 2010 revealed positive genotoxic effects of SWCNTs through *in vitro* Comet assays. Further, micronucleus assays by Migliore et al., 2010; Cveticanin et al., 2009; Di Giorgio et al., 2011 and DNA-double-strand breaks assays by Pacurari et al., 2008, Cveticanin et al., 2009 also evidenced the same.

#### Multi-Walled Carbon Nanotubes (MWCNTs)

Siegrist et al., 2014 studied the genotoxicity of MWCNTs at different concentrations on primary and immortalized human airway epithelial cells. They concluded that brief exposure of the MWCNTs causes mitotic spindle aberrations and resulting in aneuploidy in nearly 40% of the cells. They also observed that the amount of centromere fragmentation, aneuploidy and mitotic spindle damage caused due to the exposure of MWCNTs was similar to what was often observed in carcinogenesis.

Another *in vitro* and *in vivo* study by Kato et al., 2012 demonstrated that MWCNTs have genotoxic potential, evident from *in vitro* micronucleus assay on human lung carcinoma-A549 cells *in-vivo* Comet [Male ICR mice and guanine phosphoribosyltransferase (gpt) delta mice] assays. In this study, it was evident by a significant increment in micronuclei and sister chromatid exchange frequencies and breaks in DNA strands. Moreover, the comet assay also suggested possible DNA damage. Studies by Ema et al., 2012b; Cheng et al., 2009; Fukai et al., 2018 also supported the genotoxicity effects of MWCNTs.

#### Fullerenes

Studies on fullerenes have suggested the significance of understanding the better NP’s dispersion methods and the artefacts posed by the material suspensions. Dhawan et al., 2006 reported the genotoxicity data for colloidal fullerenes. They performed a Comet assay (human lymphocytes) using colloidal fullerenes in water and found a strong correlation between the concentration of C-60 and genotoxicity. Nielsen et al., 2008 studied the genotoxic effects of a mixture of C60 and C70 by conducting a bacterial mutation assay (*Salmonella typhimurium* TA100, TA1535, TA98 and TA1537 and *Escherichia coli* WP2uvrA/pKM101 strains) and Comet assay (human lymphocyte). However, they reported no direct DNA reactive effects; it may have played an essential role in ROS-mediated carcinogenesis. Several studies carried out recently by Kyzyma et al., 2019; Prylutska et al., 2019; Tao et al., 2019 had concluded the genotoxicity effects of the fullerenes and its compounds.

### Metal and Oxide Nanoparticles

Metal nanoparticles are inorganic metal/metal oxide cores covered with inorganic/organic/inorganic oxide shells. Like others,metal-based NPs also have broad applications in the cosmetics industry, drug industry, food and health care industry.

#### Silver Nanoparticles

Silver NPs have gained much attention in recent years owing to their anti-microbial properties. The mechanism behind the genotoxic effects of Ag-NPs lies in the induction of oxidative stress resulting in DNA damage. AshaRani et al. (2008) reported the genotoxicity of silver nanoparticles (starch coated) for the first time. The toxicity was evaluated for human lung fibroblast cells through Comet assay and micronuclei assay. They proposed that the Ag NPs disrupt the mitochondrial respiratory chain, resulting in Reactive Oxygen Species (ROS) production.

Further, they interact with the ATP synthesis and thereby causing DNA damage. This DNA damage was found to be dose-dependent. In a similar study, Ghosh et al. confirmed the involvement of ROS leading to DNA damage and chromosomal aberrations after exposure to AgNPs for human lymphocytes and Swiss albino male mice. Rodriguez-Garraus et al., 2020; Park et al., 2011; Foldbjerg et al., 2010; Guo et al., 2016 and many others reported a positive correlation between Ag NPs and genotoxicity.

#### Titanium Dioxide Nanoparticles

Researchers have been using several animal models and different cell lines to test for the genotoxicity effects of both coated and uncoated TiO2NPs. In this study, different TiO2NPs ranging from 0.5 to 10 mg/cm^2^ were exposed to Syrian hamster embryo fibroblasts, and clastogenic responses were noted using CREST antibodies along with micronucleus assay. Moreover, in this study, it was also suggested that the NPs interacted with the membranes of fibroblast cells resulting in the induction of ROS production, which possibly leads to lipid peroxidation, Calcium imbalance and alteration in metabolic pathway (Rahman et al., 2002).

In one study, HepG-2 cells were exposed to different concentrations of TiO2 NPs (10, 20, 50,100 mg/ml). DNA lesions and increment in micronuclei was assessed using comet assay and micronuclei assay, respectively. Moreover, the formation of DNA lesions was dose-dependent (Osman et al., 2010). A similar observation was also elucidated by another study revealing that TiO2 NPs induced oxidative DNA damage and increased micronucleus frequency even at a low concentration (1 µg/ml) in HepG2 cells (Shukla et al., 2013). Earlier, a probable mechanism behind the genotoxic behaviour of TiO2 NPs while experimenting with the human skin cells was discussed by Shukla et al., in 2011. They observed that the interaction of these nanoparticles induces oxidative stress, leading to DNA damage and micronuclei formation. The results produced by Kang et al., 2008 suggested that apart from ROS initiation, the TiO2 NPs also cause the accumulation and activation of p53 proteins.

Ghosh et al., 2012, suggested lipid peroxidation as the second cause of DNA damage. Observation of another study suggests the inflammatory reaction in peripheral blood due to changes in cytokine expression. The scholars found it to be an indirect pathway to genotoxicity (Trouiller et al., 2009). Similarly, a previous study explicitly revealed that TiO2 NPs induced oxidative stress in the mice after 14 days of consecutive exposure, triggering oxidative DNA damage that further leads to apoptosis through the intrinsic pathways. (Shukla et al., 2014).

Thus, several evidentiary studies suggest genotoxic effects of TiO2 NPs *via* induction of ROS production leading to DNA damage, but it appears to be independent of the size and form of the said NPs.

#### Zinc Oxide Nanoparticles

ZnO NPs have gained much interest due to their antibacterial activity and application in several cosmetic products. Researchers have found the accumulation of the ZnO NPs in the human liver after a brief exposure. The Comet assay performed on ZnO NPs exposed to HepG2 cell lines shows a positive correlation between oxidative stress and DNA damage resulting in apoptosis (Sharma et al., 2011).

While reviewing the toxic effects of ZnO NPs, Singh, 2019 described that the nanoparticle stimulates toxicity in three different ways: 1) Releasing Zinc ions; 2) Production of ROS; 3) directly entering the core and associating with or get cross-connected with DNA strand at the time of cell division. Earlier, Sharma et al., 2009 assessed the genotoxicity of zinc oxide NPs in a human epidermal cell line (A431). They observed the induction of oxidative stress and lipid peroxidation in the cells due to nanoparticle exposure. The comet assay showed possible DNA damage. Other studies like Heim et al., 2015, Yin et al., 2010 also demonstrated the toxic effects of ZnO NPs even at the lower concentrations.

#### Cerium Oxide Nanoparticles

Cerium is a member of rare Earth metals-lanthanide. It is very reactive with strong oxidizing power. Cerium dioxide nanoparticles (CeO_2_ NPs) are widely engineered for various applications due to their catalytic properties. Some studies have reported the radioprotective activities of the NPs. However, few have shown the strong genotoxic effect of the CeO_2_ NPs**.** Préaubert et al., 2018 was the first to report the genotoxicity of CeO_2_ nanoparticles on human spermatozoa. They found a significant amount of DNA damage as measured by Comet assay, even at low concentrations. Lee et al. conducted *in vivo* studies on *Daphnia magna* and *Chironomus riparius* and reported DNA strand breaks. Moreover, these strand breaks were higher at lower concentrations. In a similar study, Sprague Dawley rats were exposed to different concentrations of the NPs (0.15, 0.5, 1, 3.5, or 7 mg/kg). The results claimed that the NPs induced cellular toxicity, air/blood barrier damage, and phospholipidosis (Ma et al., 2010).

Currently, there are not enough studies to prove the genotoxic effects of CeO2 NPs. Still, a state of confusion persists between the genotoxic and protective effects of CeO2 NPs. More studies dedicated to understanding the genotoxic mechanism of the CeO2 NPs needs to be conducted to fill in the knowledge gap.

#### Iron Oxide Nanoparticles

Iron oxide (Fe_2_O_3_/FeO) NPs can be found in various forms such as hematite, maghemite and magnetite. Magnetic and Superparamagnetic Fe_2_O_3_/FeO NPs are collectively called Iron oxide nanoparticles (IONPs). They are widely used in drug delivery systems due to their biocompatibility. Several researchers have studied coated and uncoated NPs in the past for their genotoxic effects. Yanping Liu et al., 2014 conducted a genotoxic study of iron oxide NPs with varied particle sizes (10, 30 nm) and surface coatings (PEG, PEI) utilizing the standard assays - Ames assay, *in vivo* micronucleus assay and Comet assay. To their observation, IONPs with PEG coating showed mutagenic activity but no chromosomal and clastogenic abnormalities. Further, smaller IONPs are more mutagenic than larger ones, whereas IONPs with PEI coating were not genotoxic. They thus concluded that the mutagenic potential of IONPs depends upon their particle size and surface coating.

A positive genotoxic response was observed and was measured by Comet assay and micronucleus assay when A549 alveolar cells were treated with nano-magnetite (Könczöl et al., 2011). However, as studied earlier with other metal nanoparticles, the recent study proves that oxidative stress has a minimal role in inducing genotoxicity by surface-modified IONPs (Mesarosova et al., 2014). To summarise, all the available genotoxic data of IONPs are inconsistent. A few studies obtained positive genotoxic effects of the IONPs using the standard assays, whereas many have reported a negative response at the exact quantities.

#### Silica

Silica nanoparticles (Si NPs) are found in two varied forms: amorphous and crystalline. Various studies on both forms of silica are available for their genotoxicity**.** A detailed study using different sizes (10, 25, 50, 100 nm) of SiNPs was conducted on human umbilical vein endothelial cells (HUVECs). It was observed that the NPs were able to induce DNA damage, which is negatively correlated with the size of the NPs. A significant increase in the intercellular ROS was also observed in the cells after NPs exposure (Zhou et al., 2018). Similar effects were recorded when three epithelial cell lines-A549, HT29 and HaCaT were treated with Si NPs. In addition, single-strand breaks and alkaline labile sites were observed *via* standard Comet assay (Mu et al., 2012). Studies by Yazdimamaghani et al., 2019; Barnes et al., 2008 had also found similar genotoxic effects of the Si NPs.

#### Cobalt and Its Alloys

The genotoxic effects of cobalt ion, cobalt-chromium mixture and cobalt-iron mixed NPs have been studied well in the past. In a study assessing the genotoxic effect of cobalt NPs and Co2+ in Balb/3T3 cells, researchers found that Co. NPs are genotoxic in a dose-dependent manner (Ponti et al., 2009). The exposure of cobalt ions and cobalt NPs to human leukocytes demonstrated higher DNA damage caused by cobalt NPs than Co2+ ions. Conversely, the increased frequency of micronucleus was observed in the case of Co2+ ions. With this, the researchers thus hypothesized that the salting-out effect of Co2+ ions modulated the genotoxic effects. Further reports on genotoxic effects of cobalt alloys and cobalt-based NPs can be appraised in Colognato et al., 2007; Colognato et al., 2008; Pan et al., 2010; Wan et al., 2017.

#### Copper Oxide Nanoparticles

Not many studies have assessed the genotoxicity of CuO NPs. While measuring the DNA damage in A549 cell lines by briefly exposing them to CuO NPs, researchers found that CuO NPs were more genotoxic than TiO_2_and ZnO NPs. This implies that the oxidative behaviour of the CuO NPs is the primary mechanism behind the toxic effect of the respective NPs (Pan et al., 2010.) while, it was also observed that Cu ions were much less toxic when compared with CuO NPs (Karlsson et al., 2008). A similar study to test the mutagenicity of the CuO NPs having a size ranging more than 50 nm was conducted *via* Ames assay. The low mutagenic level was shown in TA100 strains and TA97a strains.

## Conclusion

The past decade has witnessed tremendous growth in the field of nanotoxicology. Several studies have utilized the standard test assays to understand the potential hazards posed by natural and engineered NPs *in-vivo* and *in-vitro*. Nanoparticles are now being manufactured for their wide range of applicability in biomedical science, food industry, cosmetics and several other such industries for human application.

NPs exposure to humans is inevitable. They tend to pose several genotoxic effects on organisms. The researchers are putting all their efforts into elucidating our limited knowledge and understanding about the possible effects of NPs. It is well known today that inflammatory reactions play a vital role in inducing genotoxic reactions. Conversely, there are studies with contradictions on the size-, shape- and dose-dependent effect of the NPs. Experiments with *in-vivo* and *in-vitro* models also claimed different observations when an equivalent dose of the NPs was administered. Therefore, a brief re-evaluation of the standard battery of assays is required to understand the cellular uptake of the nanoparticles, their bioaccumulation, the biological barrier-crossing mechanisms and the precise nature of the interaction between the nanoparticle and the genetic material.

## Future Considerations and Perspectives

As our knowledge unfolds about epigenetic modifications, more detailed studies on the functional consequences of interactions between NPs and the epigenetic modification elements are also essential. Several studies have already reported the possible impact of nanoparticles (NPs) on the epigenome. However, it is suggestive that the studies on the mechanisms underlying these modifications-their inheritance pattern and how these changes are restored in a cell–must be encouraged to detangle the mysteries behind. In addition to this, in future studies, micro-RNA or epigenetic mechanisms relating to gene expression must also be considered. Nevertheless, as we know that science is ever-evolving, the field of nanoparticles is still growing. Scientists are developing novel aspects of NPs by each passing hour. The fields of nanotechnology and nanotoxicology are linked together. While nanotechnology is developing an investigative approach, on the other hand, nanotoxicology is developing a preventive approach. Thus, we can say that the future of Nanotechnology and Nanotoxicology holds great promise, and one needs to consider their challenges.
